# Contributions of wheat and maize residues to soil organic carbon under long-term rotation in north China

**DOI:** 10.1038/srep11409

**Published:** 2015-06-23

**Authors:** Jinzhou Wang, Xiujun Wang, Minggang Xu, Gu Feng, Wenju Zhang, Xueyun Yang, Shaomin Huang

**Affiliations:** 1 Ministry of Agriculture Key Laboratory of Crop Nutrition and Fertilization, Institute of Agricultural Resources and Regional Planning, Chinese Academy of Agricultural Sciences, Beijing 100081, China; 2 Earth System Science Interdisciplinary Center, University of Maryland, College Park, MD 20740, USA; 3 College of Resources and Environmental Sciences, China Agricultural University, Beijing 100094, China,; 4 College of Global Change and Earth System Science, Beijing Normal University, Xinjiekouwai Street No.19, Haidian District, Beijing 100875, and Joint Center for Global Change Studies, Beijing 100875, China; 5 State Key Laboratory of Desert and Oasis Ecology, Xinjiang Institute of Ecology and Geography, Chinese Academy of Sciences, Urumqi, Xinjiang 830011, China; 6 College of Natural Resources and Environment, Northwest Agricultural and Forestry Science and Technology University, Yangling, Shaanxi 712100, China; 7 Institute of Plant Nutrition, Resources and Environment, Henan Academy of Agricultural Sciences, Zhengzhou, Henan 450002, China

## Abstract

Soil organic carbon (SOC) dynamics in agro-ecosystem is largely influenced by cropping. However, quantifying the contributions of various crops has been lacking. Here we employed a stable isotopic approach to evaluate the contributions of wheat and maize residues to SOC at three long-term experimental sites in north China. Soil samples were collected from 0–20, 20–40, 40–60, 60–80 and 80–100 cm after 13 and 20 years of wheat-maize rotation, and SOC and its stable ^13^C composition were determined. Our data showed that the δ^13^C value of SOC varied, on average, from −22.1‰ in the 0–20 cm to −21.5‰ in the 80–100 cm. Carbon input through maize residues ranged from 35% to 68% whereas the contribution of maize residues to SOC (0–40 cm) ranged from 28% to 40%. Our analyses suggested that the retention coefficient was in the range of 8.0–13.6% for maize residues and 16.5–28.5% for wheat residues. The two-fold higher retention coefficient of wheat versus maize residues was due to the differences in the quality of residues and probably also in the temperature during the growing season. Our study highlighted the importance of crop management on carbon sequestration in agricultural lands.

Soil organic carbon (SOC), a key index for soil fertility and a major carbon (C) reservoir in terrestrial ecosystems, plays an important role in crop productivity and the global C cycle^1^,^2^. Thus, enhancing C sequestration as SOC in cropland is recognized as a win-win strategy^2^. Soil organic carbon dynamics depends on the balance between C inputs (e.g., via return of crop residues and organic amendments) and C outputs (primarily through SOC decomposition). While organic C input often leads to SOC enhancement, the relationship between these two parameters is not straightforward. For example, a few studies showed a linear relationship between SOC and C input^3^,^4^, but there was also evidence of a non-linear relationship^5^,^6^.

Decomposition of SOC is largely regulated by microbial activity, which may be influenced by many factors. Among those, the quality of plant residues has direct impacts on SOC decomposition^7^,^8^. In general, the decomposition rate of plant residues is negatively related to the C:N ratio and lignin content^7^,^9^. In addition, climate and soil conditions have large effects on SOC decomposition^3^,^10^,^11^,^12^. Optimal temperature and soil moisture for microbial activity can cause high rates of residue decomposition and thus low rates of SOC accumulation^3^,^13^,^14^. On the other hand, SOC in soils with more clay tends to have a longer turnover time than in sandy soils^15^,^16^.

Recent studies have shown an increasing trend in SOC stock over the last three decades in the majority of north China’s cropland^17^,^18^. For example, Pan *et al.*^18^ reported an increase of 12.40 Tg C yr^−1^ between 1985 and 2006 in north China where wheat-maize rotation has been a dominant cropping system. There have been a few reports of positively linear relationships between SOC stock and C input through the return of wheat and maize residues in this region^3^,^19^. However, there has been limited quantification of the relative contributions of maize and wheat residues to SOC in the maize-wheat rotation system.

Because plant residue is a main contributor for SOC pool, crop type regulates the stable carbon isotopic compositions (δ^13^C) in SOC since the different isotopic signatures in assimilated CO_2_ form as a result of photosynthetic ^13^C discrimination. On average, δ^13^C value is −27‰ and −13‰ in C3 and C4 plants, respectively^20^. Thus, stable C isotopic techniques have been widely used to quantify the relative contributions of C3 and C4 plants to SOC^21^,^22^,^23^,^24^.

In this study, we analyzed data generated from three long-term experiments (LTEs) in north China, which had been under wheat-maize rotation since 1990. We obtained archived soil samples (from 0–100 cm profiles) collected in 2002 and 2009, and determined SOC and its stable ^13^C composition. Our objectives were to quantify the relative contributions of wheat and maize residues to SOC in soil profile under long-term wheat-maize rotation, and to explore the difference (and possible reasons) in the retention coefficient (i.e., the net changes of SOC per unit C input) between wheat and maize residues.

## Results

### Cumulative C input

**Figure 1:**
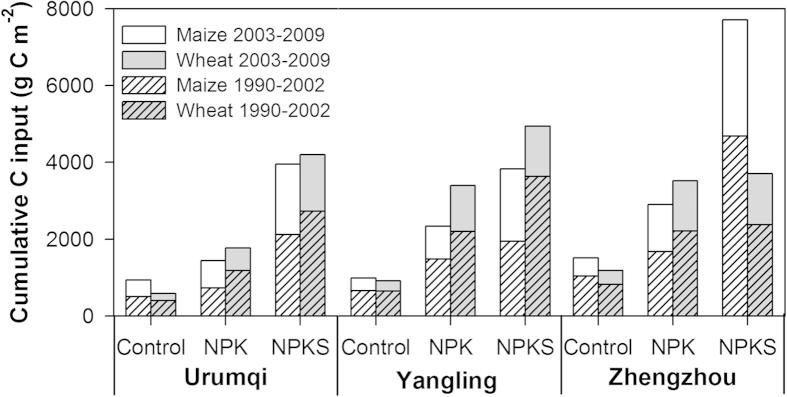
Cumulative carbon inputs by maize and wheat residues under different treatments during the periods of 1990–2002 and 2003–2009.

Cumulative C inputs for the periods of 1990–2002 and 2003–2009 are presented in Fig. 1. Clearly, the control treatment yielded the lowest C inputs of both wheat and maize residues. As expected, fertilization treatments led to a greater rate of C input than the control treatment. For example, C input through maize (wheat) residues during the period of 2003–2009 was only 330–475 (188–265) g C m^−2^ in the control, but 715–1827 (578–1471) g C m^−2^ at Urumqi with a mono-cropping system and 854–3029 (1200–1329) g C m^−2^ at Yangling and Zhengzhou with a double cropping system under fertilization treatments. For both maize and wheat residues, C input followed the same order (i.e., control < NPK < NPKS) for both periods at all the sites.

Under non-fertilization, C input was greater from maize residue compared to wheat residue. However, C input through maize residue was less than that through wheat residue under the NPK treatment. For the NPKS treatment, the C input rate through maize residue was comparable with that through wheat residue at Urumqi, but relatively lower at Yangling and much greater at Zhengzhou, due to the differences of straw return rate between sites.

### Vertical distributions of δ^13^C, SOC and maize and wheat derived SOC

**Figure 2:**
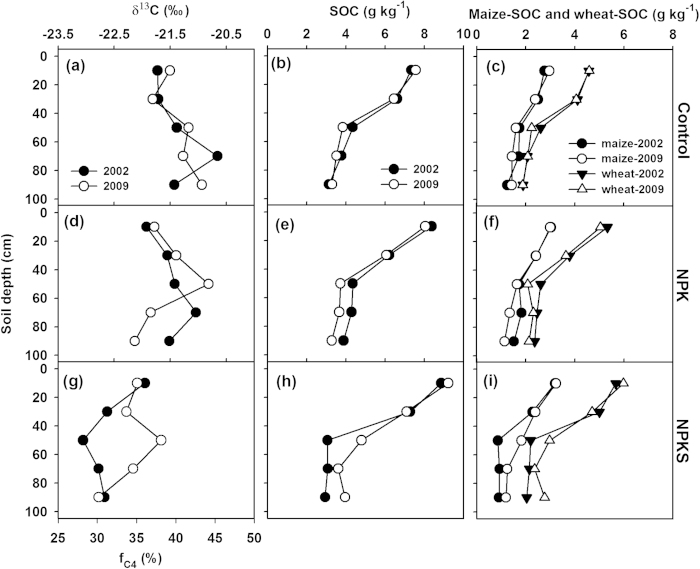
Vertical distributions of δ^13^C in SOC, fraction of maize derived SOC (f_C4_) (left column), total SOC (middle column), maize and wheat derived SOC (right column) under control (top row), NPK (middle row) and NPKS (bottom row) at Urumqi.

Figure 2 illustrated that there was a small variation in the δ^13^C value of SOC in the 0–20 cm, but a considerable variation below 20 cm at Urumqi. There was an overall similarity in the magnitude and vertical distribution in the δ^13^C value of SOC between the control and NPK, with an enrichment of ^13^C in the subsoil (~60 cm). However, the NPKS treatment showed a depletion of ^13^C below 20 cm. The estimated fraction of maize derived SOC was 37–45%, 35–44% and 28–38% for the control, NPK and NPKS, respectively. Total SOC content showed a decrease, from 7–9 g kg^−1^ in the 0–20 cm to ~4 g kg^−1^ below 40 cm. Maize and wheat derived SOC contents revealed similar vertical distributions. For the 0–40 cm, SOC stock ranged from 1342 to 1428 g m^−2^ for maize derived SOC, and from 2203 to 2722 g m^−2^ for wheat derived SOC (Table 1), with the highest found under the NPKS treatment.

**Figure 3:**
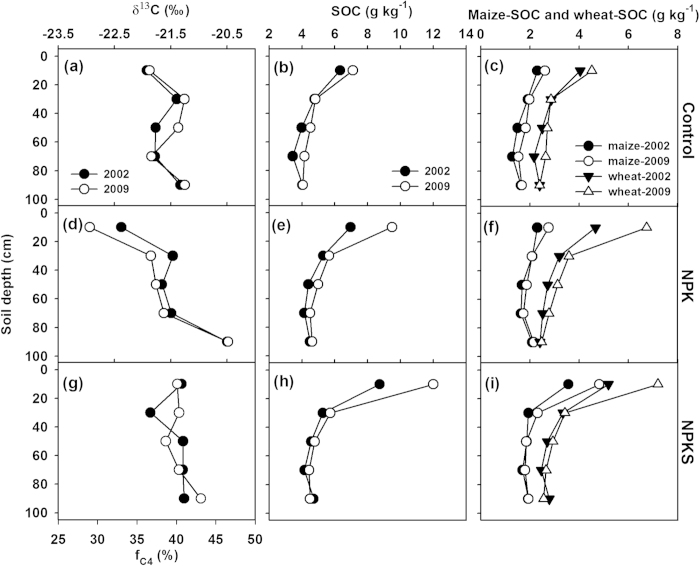
Vertical distributions of δ^13^C in SOC, fraction of maize derived SOC (f_C4_) (left column), total SOC (middle column), maize and wheat derived SOC (right column) under control (top row), NPK (middle row) and NPKS (bottom row) at Yangling.

The value of δ^13^C in SOC at Yangling showed a weaker vertical variability except under the NPK that showed a value of <−22.4‰ in the 0–20 cm and −20.5‰ in the 80–100 cm (Fig. 3). Overall, the estimated fraction of maize derived SOC was in a range of 29–47%. While there was no clear difference in the δ^13^C of SOC between 2002 and 2009, there was an increasing trend in SOC from 2002 to 2009, particularly in the 0–20 cm under fertilization treatments. Data in table 1 illustrates that in the top 40 cm, maize derived SOC increased from 1215–1563 g m^−2^ in 2002 to 1319–2021 g m^−2^ in 2009, and wheat derived SOC from 1980–2441 g m^−2^ to 2102–3008 g m^−2^.

**Figure 4:**
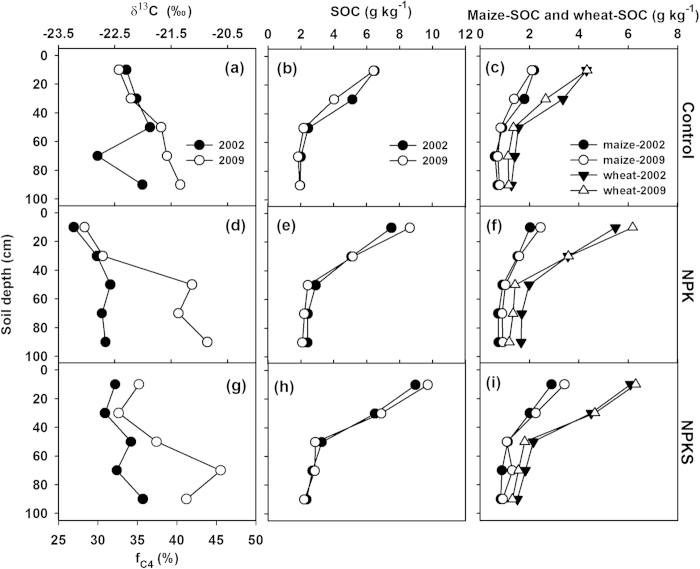
Vertical distributions of δ^13^C in SOC, fraction of maize derived SOC (f_C4_) (left column), total SOC (middle column), maize and wheat derived SOC (right column) under control (top row), NPK (middle row) and NPKS (bottom row) at Zhengzhou.

A small difference in the value of δ^13^C in SOC above 40 cm, but a large difference below 40 cm occurred at Zhengzhou with enrichment of ^13^C in 2009 (Fig. 4). On average, the estimated fraction of maize derived SOC was 27–37% and 28–46% for year 2002 and 2009, respectively. Overall, there was little change in total SOC stock over time. However, maize derived SOC stock in the 0–40 cm increased by ~15% from 1007–1394 g m^−2^ in 2002 to 1144–1611 g m^−2^ in 2009 under fertilization treatments (Table 1).

Statistical analyses showed that the δ^13^C value of SOC was significantly affected by site, year, and depth (Table S1). In particular, there were significant differences (*P* < 0.001) between sites and between depths. Table 2 shows that ^13^C in SOC of the 0–40 cm was more depleted at Zhengzhou (~ −22.5‰) compared with that at Yangling (−21.5 to −22‰) and Urumqi (~ −21.9‰), indicating smaller contribution to SOC by maize at Zhengzhou (~32%) relative to the other two sites (36–39%). ^13^C in SOC was depleted in the 0–20 cm (−22.1‰), and became enriched below 40 cm (−21.7‰). Accordingly, maize’s contribution to SOC increased from 34.8% to 39.2%.

### Comparison of maize’s contribution to C input and SOC

**Figure 5:**
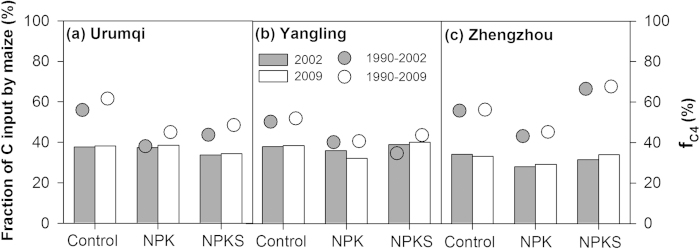
Fractions of carbon input by maize during 1990–2002 (solid circles) and 1990–2009 (hollow circle), and maize’s contribution to SOC (f_C4_, 0–40 cm) in 2002 (grey bars) and 2009 (white bars) under different fertilization treatments.

Figure 5 illustrated that the percentage of C input through maize residues varied from 35% to 68%, which was higher than maize’s contribution to SOC (28–40%) over the 0–40 cm across all three sites. There were considerable differences in the percentage of C input through maize residues but relatively small differences in the maize’s contribution to SOC between treatments. For instance, the percentage of C input through maize residues was significantly higher under the control treatment than under the NPK at all three sites. However, maize’s contribution to SOC was slightly higher under the control treatment relative to the NPK treatment only at Zhengzhou and Yangling. Although the percentage of C input through maize residues at Urumqi was greater under the control (56–62%) than under the NPK treatment (38–45%), maize’s contribution to SOC was the same under the two treatments (~38%). Similarly, C input through maize residues was much greater under the NPKS treatment (66–68%) relative to the control treatment (56%) at the Zhengzhou site, while maize derived SOC was similar (~33%) under both treatments. On average, the percentage of C input through maize residues was 48–51% whereas maize’s contribution to SOC was 35% over the 0–40 cm, indicating that the retention coefficient of maize residues was lower than wheat residues under long-term wheat-maize rotation in northern China.

## Discussion

Our data showed that ^13^C became enriched in SOC with depth (Fig. 2, 3, 4, Table 2), which was consistent with the typical trends observed in well-drained soils under C3 or C3/C4 mixed vegetation^21^,^24^,^25^,^26^. The depletion of ^13^C in the topsoil (with newer crop residues) might partly reflect the trend of δ^13^C in the atmospheric CO_2_. There is evidence that the δ^13^C values of both wheat and maize tissues decreased over the past decades^27^,^28^, which would lead to the depletion of ^13^C in SOC. On the other hand, the enrichment of ^13^C in the subsoil (with older SOC) might be partly caused by the isotopic fractionation during SOC decomposition^25^,^29^. In addition, subsoil was less responsive to changes in land use^26^,^30^.

In agricultural ecosystem, management practices, e.g., fertilization and straw retention, could have effects on the δ^13^C value in SOC^19^,^23^,^31^. Our study demonstrated that fertilization treatments mainly affected the δ^13^C of SOC in the top layer (Fig. 2, 3, 4). For example, at Yangling and Zhengzhou, NPK treatment resulted in more depletion of ^13^C in top layer due to its relatively lower C input through maize residues, compared with the control and NPKS treatment. However, at Urumqi, although C input through maize residues was greater without fertilization than the NPK treatment, the δ^13^C values of SOC were similar. This was probably due to the small amount of crop residues returned into soil under non-fertilization at Urumqi (Fig. 1), which might require a longer period to show significant effects on the δ^13^C value in SOC.

**Figure 6:**
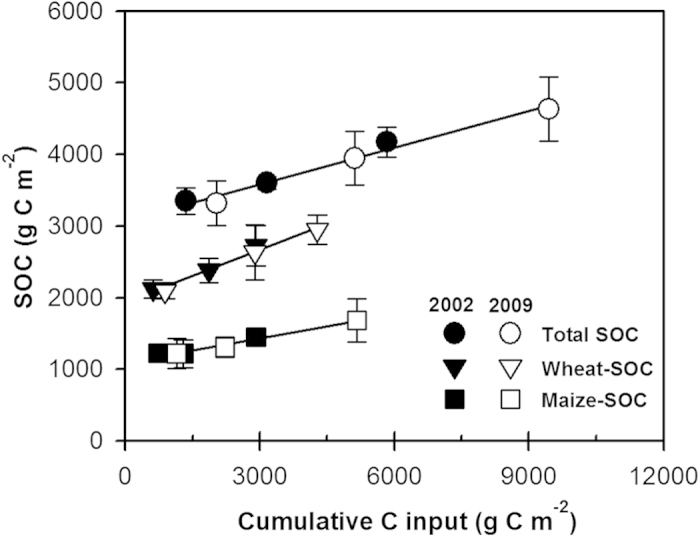
Relationships between SOC stocks in 0–40 cm for 2002 (2009) and cumulative C inputs during 1990–2002 (1990–2009), using the averages of all sites. The error bars denote standard deviations. Maize-SOC: y = 0.112x + 1102, R^2^ = 0.97; Wheat-SOC: y = 0.244x + 1937, R^2^ = 0.98; Total SOC: y = 0.170x + 3073, R^2^ = 0.98.

Based on the linear relationship between SOC stock over the 0–40 cm and cumulative C input, one could estimate the retention coefficient of crop residues, i.e., the slope of the regression line. As illustrated in Fig. 6, the retention coefficient was 24.4% and 11.2% for wheat and maize residues, respectively, and 17.0% for the wheat-maize rotation system.

Due to the difficulty of direct measurement of roots and a lack of data for δ^13^C values in wheat and maize, we estimated root biomass by multiplying aboveground biomass with root:shoot ratios and used mean δ^13^C values for wheat and maize residues from literature^32^, which might introduce some uncertainties in our calculations of retention coefficient. As such we conducted uncertainty analyses by testing different values for root:shoot ratio and δ^13^C in wheat and maize residues. Table 3 summarises comparisons of the reference method based on Bolinder *et al.*^32^, with three other commonly used methods by setting the root:shoot ratios to: (I) 0.24 and 0.22 (i.e., IPCC2006)^33^, (II) 0.43 and 0.35 (i.e., Li1994)^34^, and (III) 0.50 and 0.33 (i.e., BW1997)^35^ for wheat and maize, respectively. On average, the maize:wheat ratio in C input was higher in the reference (1.27 ± 0.49) than in the IPCC2006 (1.04 ± 0.44), Li1994 (1.02 ± 0.37) and BW1997 (0.88 ± 0.32). Interestingly, the retention coefficient of maize residues was similar (varying from 10.5% to 12.1%) across all methods whereas the retention coefficient of wheat residues varied considerably, from 16.5% to 24.6%. The uncertainty associated with the chosen δ^13^C values for wheat and maize was small, in which the retention coefficient was in a range of 8.0–13.6% for maize and 21.4–28.5% for wheat (Table 4).

Our estimated retention coefficient of maize residues was close to those (5.3–16.6%) observed in the Corn Belt region of the central USA under 8–35 years of continuous maize cropping^21^,^22^. Similarly, Kristiansen *et al.*^31^ reported a retention coefficient of 11–15% for maize straw in four Danish arable soils. It seems that our estimated retention coefficients of wheat residues were higher than some earlier reports. For instance, Follett *et al.*^24^ reported a value of 5.4% following 84 years of wheat-fallow cultivation at Akron and 10.5% after 20 years of cropping at Sidney in the North American Great Plains. These variations in retention coefficient were probably due to the differences in climate and soil conditions and the duration as well.

Despite some differences in the findings from the limited reports, there were studies showing greater contribution to SOC by wheat residues than maize residues. For example, based on a 16 year continuous cropping experiment (conducted in a semi-arid, subtropical highlands of Central Mexico), Fuentes *et al.*^23^ demonstrated that the contribution of maize residues was significantly smaller than that of wheat residues. There was also other evidence of relatively faster decomposition of maize residues than wheat residues, based on a two-year field incubation at Sanborn Field in the Midwest US^35^.

There were a few field incubation experiments (using buried bag methods) of comparing C remaining or retention between maize and wheat straws and/or roots in China^10^,^36^,^37^. Table 5 illustrates that all studies reveal a decreasing trend in the remaining of crop straw/root with time. Clearly, root has more C remaining thus higher retention coefficient than straw for both maize and wheat^37^. In addition, studies by Liu *et al.*^36^ and Wang *et al.*^37^ indicated that the retention coefficient of maize straw:root was significantly lower than that of wheat, which yielded the maize:wheat ratio in retention coefficient ranging from 0.53 to 0.77. On the other hand, Wang *et al.*^10^ demonstrated that decomposition of maize straw was more sensitive to environmental changes than that of wheat straw during 1–2 years of incubation, which resulted in a large variation (0.52–1.22) in the maize:wheat ratio of retention coefficient.

There may be many driving factors responsible for the differences in the retention between maize and wheat residues. A few studies have demonstrated that the decomposition rate of litter was negatively related to the C:N ratio and lignin content in litter^7^,^8^. It appears that crop residues with higher C:N ratio tend to have a greater retention coefficient (see Fig. S1). In general, maize residues have lower lignin contents^35^,^37^,^38^ and smaller C:N ratios^39^, implying a greater decomposition rate relative to wheat residues.

Apart from the quality of residues, decompositions of maize and wheat residues may have different responses to climate conditions^10^. In our study wheat and maize have different growing seasons with their own environmental conditions that may also lead to differences in the fates of their residues. For example, the cumulative effective temperature, the sum of daily temperate above 0 °C, is greater during maize’s growing season (2889–3232 °C) than during wheat’s (1865–2603 °C) (see Table 6). Therefore, the organic materials generated from the root system of maize would be subject to a faster decomposition rate than those of wheat, resulting in lower retention coefficient for the former than for the latter.

## Materials and methods

### Site descriptions and experimental design

The National Long-term Monitoring Network of Soil Fertility and Fertilizer Effects was established around 1990 to study the responses of crop yields and soil fertility to fertilization management in the main agricultural regions of China. We used three experimental sites located in north China with arid and semi-arid climatic conditions (Table 6). The Urumqi site had a mono-cropping system with a rotation of maize - spring wheat - winter wheat whereas the other two sites had a double-cropping system with a winter wheat-summer maize rotation. All three sites had a history of cropping with various C3 and C4 rotations, in which wheat and maize were dominant crops. According to an earlier report^19^, the δ^13^C value in SOC of the 0–20 cm was −21.7‰ at Yangling and −22.2‰ at Zhengzhou prior to the experiments, reflecting a mixture of C3 and C4 cropping. Annual average temperature varied from 7.3 °C at Urumqi to 14.7 °C at Zhengzhou. Annual precipitation was generally low (299–641 mm), but open pan evaporation ranged from 1292 mm to 2015 mm. Over 60% of the precipitation occurred during the maize growing season. Irrigation was applied when needed, ranging from 225 mm at the Zhengzhou site to 450 mm at the Urumqi site. Conventional tillage was applied and ploughing depth varied from 20 cm to 30 cm. All three sites had calcareous soils, with initial SOC content in a range of 6.3–8.8 g kg^−1^, initial TN 0.67–0.87 g kg^−1^, and soil pH (in 1:2.5 soil/water) 8.1–8.6.

Three common management treatments were selected: (i) control- no fertilizer application, (ii) NPK- mineral nitrogen (N), phosphorus (P) and potassium (K) fertilization, and (iii) NPKS- mineral NPK fertilization with straw incorporation. Mineral N, P and K fertilizers used were urea, calcium superphosphate, and potassium sulphate (or potassium chloride), respectively. Details of fertilizer application were reported previously^3^,^40^. Briefly, the application rates of mineral fertilizers ranged from 123 to 242 kg N ha^−1^, 25 to 60 kg P ha^−1^, and 39 to 78 kg K ha^−1^ for each growing season (Table S2).

The aboveground biomass was removed for all the treatments, except the NPKS treatment that had straw incorporation of one crop each year, i.e., either wheat or maize at the Urumqi site and only maize at the Yangling and Zhengzhou sites. On average, annual rates of straw returned were 7, 4 and 6 Mg ha^−1^ yr^−1^ (air-dry matter) at Urumqi, Yangling and Zhengzhou, respectively, during the period of 1990–2009.

### Estimation of C input from wheat and maize residues

Crop residues included straw, stubble and roots. The amount of straw return in the NPKS treatment was measured annually. Carbon input (g C m^−2^) of stubble (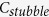) and roots (*C*_*roots*_) were determined as:


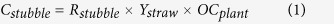



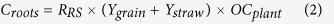


where
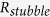
was the ratio of the stubble left in soil to the straw biomass;*R*_*RS*_ was the ratio of belowground biomass to aboveground biomass, or the root:shoot ratio; *Y*_*grain*_ and 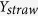 were the yields (g m^−2^) of crop grain and straw, respectively. Following Zhang *et al.*^3^, 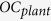, the C content (g C g^−1^) of aboveground biomass, was set as the China’s average, i.e., 0.399 and 0.444 g C g^−1^ (oven-dried basis) for wheat and maize, respectively^39^. We set *R*_*bg*_ as 0.24 and 0.29 for wheat and maize, respectively, based on Bolinder *et al.*^32^. According to Wang *et al.*^41^, *R*_*stubble*_ was 0.03 for maize, and 0.26 (0.35) for wheat with (without) fertilization treatments.

### Soil sampling and analyses

Soil samples were collected after harvest (i.e., during September-October), with 15 soil cores (5-cm-diam) collected as a composite sample from each plot from 0–20, 20–40, 40–60, 60–80 and 80–100 cm layers. Soils from the same layer were air dried and thoroughly mixed. Representative sub-samples were crushed to 0.25 mm. For SOC measurement, 1 g soil was pretreated with 10 ml 1 M HCl for 12 hours to remove carbonate. The pretreated sample was combusted at 1020 °C with a constant helium flow carrying pure oxygen to ensure completed oxidation of organic materials. Production of CO_2_ was determined by a thermal conductivity detector using a CNHS-O analyzer (Model Euro EA 3000). Stable ^13^C isotope was determined by measuring the isotopic composition of collected CO_2_ using a Finnigan MAT Delta Plus XP Isotope Ratio Mass Spectrometer at the Nanjing Institute of Geology and Paleontology, Chinese Academy of Sciences (CAS). We reported isotopic data in delta notation relative to the Vienna Pee Dee Belemnite (VPDB).

### Calculation of contributions of wheat and maize to SOC

For each layer, SOC stock was calculated from bulk density and C content. Because bulk density was only measured for the 0–20 and 20–40 cm layers (Table 6), we used the bulk density values from the 20–40 cm layer for the calculations of SOC stocks below 40 cm.

Relative contributions (%) of wheat (*f*_*C3*_) and maize (*f*_*C4*_) to SOC were calculated using a two end-member mixing model according to Balesdent *et al.*^42^:


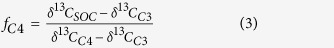



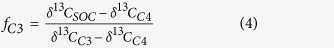


where δ^13^C_*SOC*_, δ^13^C_C3_ and δ^13^C_C4_ were the stable ^13^C composition in SOC, wheat and maize, respectively. The mean δ^13^C values for wheat and maize were approximately −27‰ and −13‰, respectively^20^,^43^.

### Statistical analyses

We carried out analyses of variance (ANOVA) to evaluate the effects of site, year, treatment, depth, and their interactions on the δ^13^C value of SOC. We applied Fisher’s protected least significant difference (LSD) for the multiple comparisons (i.e., between sites and layers). These analyses were performed by SAS 9.2 (SAS Institute, Cary, NC, USA).

## Additional Information

**How to cite this article**: Wang, J. *et al.* Contributions of wheat and maize residues to soil organic carbon under long-term rotation in north China. *Sci. Rep.* **5**, 11409; doi: 10.1038/srep11409 (2015).

## Supplementary Material

Supplementary Information

## Figures and Tables

**Table 1:** Stocks (g C m^−2^) of maize-, wheat-derived and total SOC in the 0–40 cm depth.

Treatment	Urumqi		Yangling		Zhengzhou		Mean (S.D.)*	
	2002	2009	2002	2009	2002	2009	2002	2009
Maize-derived SOC								
Control	1342	1363	1215	1319	1128	988	1228 (107)	1223 (205)
NPK	1387	1381	1273	1393	1007	1144	1222 (195)	1306 (140)
NPKS	1387	1428	1563	2021	1394	1611	1448 (99)	1686 (304)
Wheat-derived SOC								
Control	2215	2206	1980	2102	2175	1982	2123 (125)	2097 (112)
NPK	2315	2203	2257	2936	2570	2773	2381 (167)	2637 (385)
NPKS	2722	2717	2441	3008	3011	3111	2725 (285)	2945 (204)
Total SOC								
Control	3557	3569	3195	3421	3303	2969	3352 (186)	3320 (312)
NPK	3701	3584	3531	4330	3578	3917	3603 (88)	3943 (374)
NPKS	4109	4145	4004	5028	4405	4721	4173 (208)	4631 (448)

^*^Standard deviation.

**Table 2:** Average values of δ^13^C in SOC for each layer at the three LTE sites.

Layer	Urumqi	Yangling	Zhengzhou	Mean
0–20	−21.83 Aa*	−21.97 Ba	−22.59 Bb	−22.13 (34.8)**C
20–40	−21.90 Aa	−21.53 Ba	−22.49 Bb	−21.97(35.9) BC
40–60	−21.58 Aa	−21.57 Ba	−21.87 Aa	−21.68 (38.0) AB
60–80	−21.63 Aa	−21.56 Ba	−21.93 Aa	−21.71 (37.8) AB
80–100	−21.91 Ab	−20.96 Aa	−21.68 Ab	−21.52 (39.2) A

^*^Values followed by the same letter (lower case letter within a row or upper case letter within a column) are not significantly different at P ≤ 0.05 based on LSD test.

^**^Percentage of maize derived SOC

**Table 3:** Uncertainty analyses of the maize:wheat ratios in C input and retention coefficient using different root:shoot ratios (R_RS_)*.

Method	Maize:wheat in C input**		Retention coefficient		
		Maize	Wheat	Maize:wheat	
Reference		0.112	0.244	0.46
IPCC2006	1.27 ± 0.49	0.121	0.246	0.49
Li1994	1.04 ± 0.44	0.105	0.182	0.58
BW1997	1.02 ± 0.37	0.107	0.165	0.65

^*^R_RS_ values for wheat and maize were set to 0.24 and 0.29 in the reference^32^, 0.24 and 0.22 in the IPCC2006^33^, 0.43 and 0.35 in the Li1994^34^, and 0.50 and 0.33 in the BW1997^35^ methods.

^**^Mean ± standard deviation.

**Table 4:** Uncertainty analyses of the retention coefficient using different combinations of δ^13^C values (‰) for maize and wheat.

	δ^13^C		Retention coefficient		
	Maize	Wheat	Maize	Wheat	Maize: Wheat
Reference	−13	−27	0.112	0.244	0.46
S1	−13	−29	0.136	0.214	0.64
S2	−13	−25	0.080	0.285	0.28
S3	−11	−27	0.098	0.259	0.38
S4	−15	−27	0.130	0.224	0.58

**Table 5:** Remaining fraction of carbon or dry weight from wheat and maize residues after 1-2 years of field incubation.

Location	Incubation time (yr)	Maize		Wheat		Maize:wheat	
		Straw	Root	Straw	Root	Straw	Root
Beijing^a^ (39°57′N, 116°19′E)	1	0.215	0.23	0.278	0.382	0.77	0.60
	2	0.177	0.185	0.241	0.347	0.73	0.53
Hailun^b^ (47°26′N, 126°38′E)	1		0.391		0.549		0.71
	2		0.313		0.42		0.75
Hailun^c^ (47°26′N, 126°38′E)	1	0.537		0.441		1.22	
	2	0.300		0.245		1.22	
Fengqiu^c^ (35°00′N, 114°24′E)	1	0.235		0.353		0.67	
	2	0.123		0.234		0.52	
Yingtan^c^ (28°15′N, 116°55′E)	1	0.319		0.295		1.08	
	2	0.199		0.234		0.85	

^a^Values were the fraction of carbon remaining in Wang *et al.*^37^;

^b^Values were the fraction of carbon remaining in Liu *et al.*^36^;

^c^Values were the fraction of mass remaining in Wang *et al.*^10^.

**Table 6:** Locations, climate conditions^a^ and initial surface soil properties (in 1990) at the long-term experiment sites.

Variables		Urumqi	Yangling	Zhengzhou
Latitude	N	43°49´	34°17´	34°46´
Longitude	E	87°36´	108°03´	113°39´
Altitude	/ m	600	523	59
Annual mean temp.	/ ^o^C	7.3	13.5	14.7
Cum. E.T^b^ for winter wheat	/ ^o^C	2603	2115	2400
Cum. E.T for spring wheat	/ ^o^C	1865	—	—
Cum. E.T for maize	/ ^o^C	3232	2889	3056
Annual precipitation	/ mm	299	585	641
Precip. for winter wheat	/ mm	248	216	213
Precip. for spring wheat	/ mm	126	—	—
Precip. for maize	/ mm	172	369	427
Annual mean irrigation^c^	/ mm	450	270	225
Annual open pan evaporation	/ mm	2015	1292	1808
Cropping system		Mono	Double	Double
Crop rotation		Wheat-maize^d^	Wheat-maize	Wheat-maize
Plot size	/ m^2^	468	196	400
Soil classification (FAO)		Haplic Calcisol	Calcaric Regosol	Calcaric Cambisol
Parent material		Limestone	Loess	River Alluvium
Clay (<0.002 mm)	/ %	20.4	21.0	10.1
Silt (0.002–0.05 mm)	/ %	44.0	73.6	57.3
Sand (>0.05 mm)	/ %	35.6	5.4	32.6
Soil pH		8.1	8.6	8.3
Bulk density (0–20 cm)	/ g cm^−3^	1.21	1.35	1.41
Bulk density (20–40 cm)	/ g cm^−3^	1.35	1.56	1.44
SOC (0–20 cm)	/ g kg^−1^	8.8	7.44	6.7
TN (0–20 cm)	/ g kg^−1^	0.87	0.93	0.67

^a^Data are means over 1981–2010 from the China meteorological sharing service system ( http://cdc.cma.gov.cn/).

^b^Cum. E.T: cumulative effective temperature above 0 °C.

^c^Based on Zhao *et al.*^40^.

^d^Crop rotation at Urumqi during 1990–2009 was as follows: maize in 1990, 1993, 1996, 2000, 2003, 2005 and 2008; spring wheat in 1991, 1994, 2002 and 2006; winter wheat in 1992, 1995, 1997, 1998, 2001, 2004, 2007 and 2009; and cotton in 1999.
