# Supplementary material for: Contributions of wheat and maize residues to soil organic carbon under long-term rotation in north China
Source: Sci Rep. 2015 Jun 23;5:11409. doi: 10.1038/srep11409 (PMC4650633; doi:10.1038/srep11409)
Supplement: Supplementary Information [file srep11409-s1.pdf]

Contributions of wheat and maize residues to soil organic carbon under long-term rotation in north China

Jinzhou Wang[1,2,3], Xiujun Wang[4,2,5 *], Minggang Xu[1], Gu Feng[3], Wenju Zhang[1], Xueyun Yang[6], Shaomin Huang[7]

[1]Ministry of Agriculture Key Laboratory of Crop Nutrition and Fertilization, Institute of Agricultural Resources and Regional Planning, Chinese Academy of Agricultural Sciences, Beijing 100081, China,

[2]Earth System Science Interdisciplinary Center, University of Maryland, College Park, MD 20740, USA,

[3]College of Resources and Environmental Sciences, China Agricultural University, Beijing 100094, China,

[4]College of Global Change and Earth System Science, Beijing Normal University, Xinjiekouwai Street No.19, Haidian District, Beijing 100875, and Joint Center for Global Change Studies, Beijing 100875, China,

[5]State Key Laboratory of Desert and Oasis Ecology, Xinjiang Institute of Ecology and Geography, Chinese Academy of Sciences, Urumqi, Xinjiang 830011, China

[6]College of Natural Resources and Environment, Northwest Agricultural and Forestry Science and Technology University, Yangling, Shaanxi 712100, China,

[7]Institute of Plant Nutrition, Resources and Environment, Henan Academy of Agricultural Sciences, Zhengzhou, Henan 450002, China.

[*] **Correspondence:** X.J.W. (wwang@essic.umd.edu)

Supplementary Table and Figure

Table S1 Analysis of variance for $\delta^{13}C$ value of SOC

Source of variation	Degree of freedom	Sum of squares	Mean of squares	F value	P value
Site	2	5.35	2.68	13.64	<0.001
Year	1	1.63	1.63	8.29	0.005
Treatment	2	1.07	0.53	2.73	0.072
Depth	4	4.36	1.09	5.55	< 0.001
Site*treatment	4	6.10	1.53	7.78	< 0.001
Site*depth	8	3.20	0.40	2.04	0.055
Residual	68	13.34	0.20		
Total	89	35.05			

Table S2 Amounts of nitrogen, phosphorus, and potassium applied for each crop (after Zhao *et al.* [40])

Treatments	Urumqi	Yangling		Zhengzhou	
	Wheat/maize	Maize	Wheat	Maize	Wheat
		Nitrogen / kg N ha^{-1} yr^{-1}			
CK	0	0	0	0	0
NPK	242	188	165	188	165
NPKS	217+29[a]	188	165+43[a]	188	123+42[a]
		Phosphorus / kg P ha^{-1} yr^{-1}			
CK	0	0	0	0	0
NPK	60	25	58	41	36
NPKS	51+5[a]	25	58+4[a]	41	36+8[a]
		Potassium / kg K ha^{-1} yr^{-1}			
CK	0	0	0	0	0
NPK	47	78	69	78	68
NPKS	39+54[a]	78	69+57[a]	78	68+86[a]

[a] The amount was from crop straw.

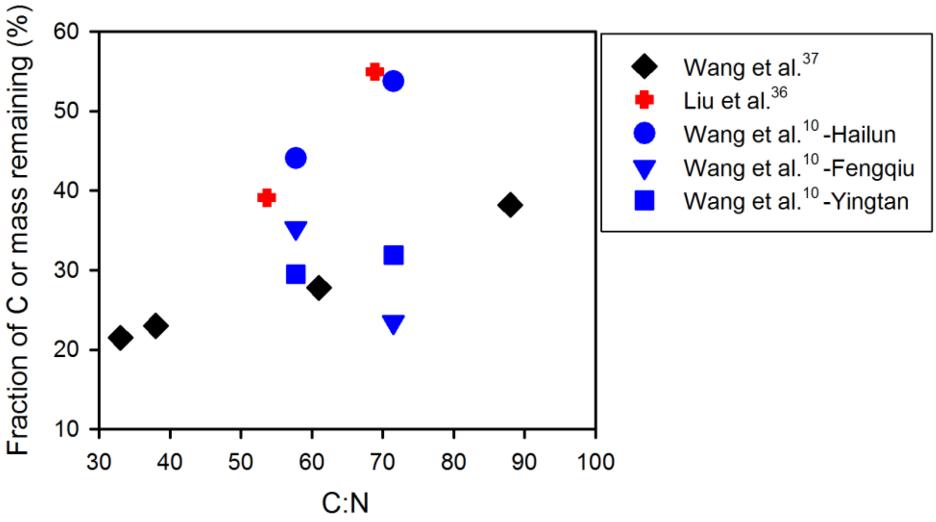

Figure S1 Relationships between the initial C:N for wheat and maize residues and the fraction of carbon or dry mass remaining after one year of field incubation
